# Mortality and toxicity of a commercial formulation of cypermethrin in *Physalaemus gracilis* tadpoles

**DOI:** 10.1038/s41598-023-45090-7

**Published:** 2023-10-19

**Authors:** Natani Macagnan, Camila Fatima Rutkoski, Alexandre Folador, Vrandrieli Jucieli Skovronski, Caroline Müller, Paulo Afonso Hartmann, Marilia Hartmann

**Affiliations:** Ecology and Conservation Laboratory, Federal University of Fronteira Sul, Erechim, RS 99.700-000 Brazil

**Keywords:** Environmental sciences, Environmental impact

## Abstract

This study evaluated the lethal, sublethal, and toxic of a commercial formulation of cypermethrin in the anuran species *Physalaemus gracilis*. In the acute test, concentrations of 100–800 μg L^−1^ were tested over 96 h. In the chronic test, cypermethrin concentrations recorded in nature (1, 3, 6, and 20 μg L^−1^) were tested for mortality and then used for the micronucleus test and erythrocyte nuclear abnormalities over a 7-days period. The LC_50_ determined for *P. gracilis* for the commercial cypermethrin formulation was 273.41 μg L^−1^. In the chronic test, a mortality of more than 50% was observed at the highest concentration (20 μg L^−1^), as it caused half of the tadpoles studied to die. The micronucleus test showed significant results at concentrations of 6 and 20 μg L^−1^ and recorded the presence of several nuclear abnormalities, indicating the genotoxic potential of the commercial cypermethrin formulation for *P. gracilis*. Cypermethrin presented a high risk to the species, indicating that it has the potential to cause several problems in the short and long term and to affect the dynamics of this ecosystem. Therefore, it can be concluded that the commercial formulation of cypermethrin had toxicological effects on *P. gracilis*.

## Introduction

Aquatic fauna is frequently exposed to pesticides due to the constant expansion of agricultural activities and intensive use for pest control^1,2^. Contamination of water resources near agricultural lands can affect the development and survival of non-target organisms, such as amphibians^3–7^.

Amphibians has been gaining importance toxicity bioassays for de evaluation of envirolmental matrices^8,9^. Anuran amphibians are considered good bioindicators of environmental pollutants due to their distinctive characteristics such as complex life cycles, rapid larval growth rates, trophic position, permeable skin^10,11^, water-dependent reproduction^12^ and unprotected eggs^11,13,14^. *Physalaemus gracilis*, popularly known as the weeping frog, has been shown to be a bioindicator species for pesticide contamination^4–7,15^. The species occurs in Argentina, Uruguay, Paraguay, and Brazil^16^ in lentic waters, protected areas, or areas of environmental change^17^ and is considered stable by the IUCN due to its wide distribution and tolerance to a wide range of habitats^18^.

Global insecticide use reached 600,000 tons in 2020, with Brazil being the third largest consumer, behind the United States, which together account for 48% of total global consumption^19^. Cypermethrin is one of the most widely used pyrethroids for agricultural and domestic applications such as cotton crops, peanuts, rice, potatoes, coffee, onions, citrus, peas, beans, snap beans, tobacco, cassava, watermelon, millet, corn, cucumber, cabbage, soybean, sorghum, tomato and tobacco^20^. With the increased use of this insecticide in agricultural areas, the likehood of contamination in nearby water bodies increases^21,22^. Cypermethrin ((RS)-α-cyano-3phenoxybenzyl (1RS,3RS;1RS,3SR)-3-(2,2-dichlorovinyl)-2,2-dimethylcyclopropane carboxylate) is classified as highly toxic (class II)^20^.

Sublethal effects reported in amphibians due to exposure to cypermethrin include behavioral, morphological, and biochemical changes in tadpoles^23–25^, mortality and alteration of metamorphosis duration, enzymatic changes, decrease in hatching success^24,25^, hyperactivity^26^, inhibition of cholinesterase activity^27^, and changes in swimming activity^7,28^. However, there is limited research addressing the genotoxic effects of cypermethrin in amphibians. Therefore, it is important to evaluate the susceptibility of anuran species to cypermethrin.

Environmental contaminations affect normal growth and development of amphibians, but induction of genetic DNA damage following exposure to pesticides is ultimately the most important adverse effect^13^. Analysis of blood cell morphology is an important bioindicator of pollution and toxic potential of substances to wildlife species^29^. Micronucleus test is one of the most commonly used methods to detect genotoxicity of chemical substances in the environment^30^. It is a rapid, effective, and inexpensive method and a good indicator of chemical contamination in organisms such as amphibians^31,32^ and can provide information on exposure to genotoxic contaminants^33^.

The objective of this study was to evaluate the toxic potential of a commercial formulation of cypermethrin in *Physalaemus gracilis* tadpoles using the micronucleus test and an ecological risk assessment.

## Results

### Mortality

**Figure 1:**
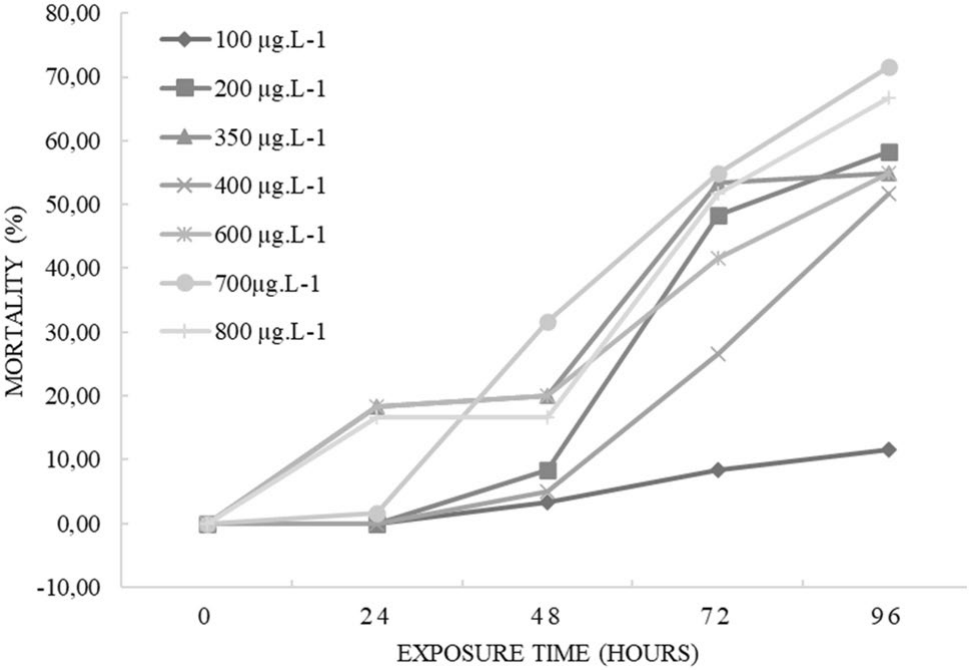
Cumulative mortality (%) of *P. gracilis* tadpoles exposed to different concentrations of the commercial formulation of cypermethrin during the acute test.

**T**he commercial formulation of cypermethrin studied showed an LC_50_ of 273.41 µg L^−1^ (− 95% = 211.22 µg L^−1^; + 95% = 353.90 µg L^−1^) for *Physalaemus gracilis* tadpoles in 96 h exposure. Tadpole mortality was significant compared to exposure time in 72 h (F_3;24_ = 5.25, *p* = 0.006; Tukey *p* = 0.043), indicating that the longer the exposure time, the greater the mortality, but not compared to concentration (F_6;35_ = 2.15, *p* = 0.07) (Fig. 1).

**Figure 2:**
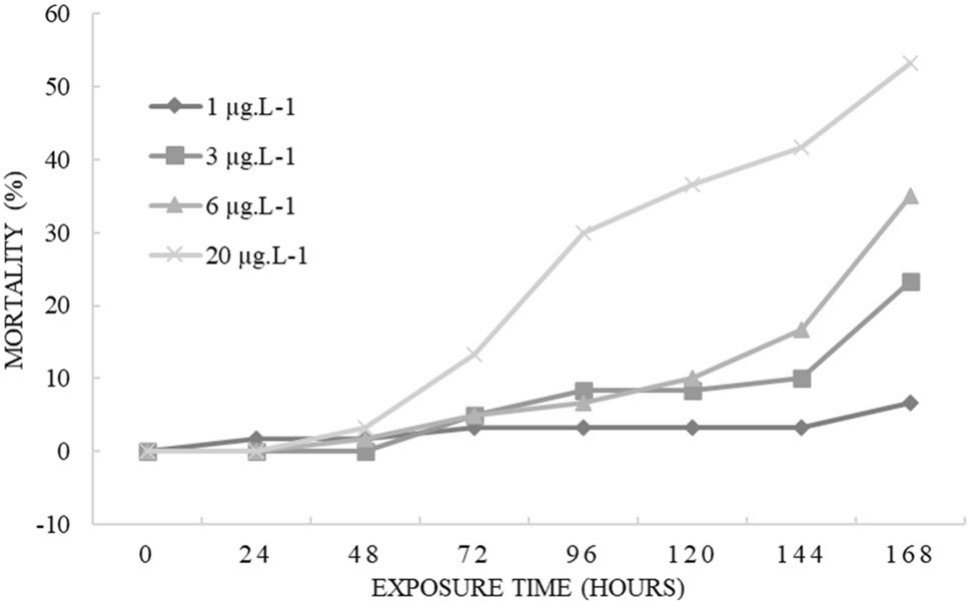
Cumulative mortality (%) of *P. gracilis* tadpoles exposed to different concentrations of the commercial formulation of cypermethrin during the chronic test.

In the chronic test, mortality of *P. gracilis* tadpoles was significant compared to concentrations 6 µg L^−1^ (F_3;20_ = 9.35, *p* = 0.026) and 20 µg L^−1^ (F_3;20_ = 9.35, *p* = 0.0004); and compared to time after 168 h (F_6;21_ = 2.97, *p* = 0.02) (Fig. 2).

### Micronucleus and erythrocyte nuclear abnormalities

The increase in micronuclei was significant in individuals exposed to concentrations of 6 µg L^−1^ (F_4,70_ = 7.72; *p* < 0.01) and 20 µg L^−1^ (F_4,70_ = 7.72; *p* < 0.01) compared to the control treatment (Table 1). The concentration of 20 µg L^−1^ promoted 4 times more micronuclei compared to the control treatment.

**Table 1:** Micronucleus and erythrocyte nuclear abnormalities (ENAs) in *P. gracilis* tadpoles exposed to various concentrations of the commercial formulation of cypermethrin for 168 h.

ENAs	Concentration (µg L^−1^)				
	Control	1	3	6	20
Micronucleus	0.46 ± 0.83 (0–3)	0.33 ± 0.62 (0–2)	0.46 ± 0.74 (0–2)	1.33 ± 0.90 (0–3)*	1.87 ± 1.40 (0–5)*
Anucleated	0	0.33 ± 0.49 (0–1)	0.07 ± 0.26 (0–1)	0.07 ± 0.26 (0–1)	0.13 ± 0.35 (0–1)
Apoptosis	0.47 ± 0.52 (0–1)	3.93 ± 6.28 (0–25)	1.67 ± 3.87 (0–15)	1.67 ± 1.99 (0–7)	11.87 ± 8.75 (1–33)**
Binucleated	0.67 ± 0.97 (0–3)	0.93 ± 1.33 (0–5)	0.53 ± 0.83 (0–3)	0.4 ± 0.63 (0–2)	0.6 ± 0.98 (0–3)
Bubble	6.13 ± 4.29 (0–13)	5.07 ± 3.15 (1–14)	3.73 ± 3.45 (0–10)	13.3 ± 0.06 (4–25)**	25.3 ± 9 (11–40)**
Karyolysis	0	0.13 ± 0.35 (0–1)	0	0.07 ± 0.26 (0–1)	0.33 ± 1.05 (0–4)
Notched	35 ± 12.69 (16–53)	32.27 ± 11.36 (13–56)	54.13 ± 22.46 (11–86)	77.8 ± 31.49 (34–127)*	104.07 ± 27.40 (60–160)*
Lobed	13.53 ± 7.96 (2–25)	7.33 ± 5.12 (1–17)	27.67 ± 15.60 (5–52)**	20.8 ± 14.62 (8–60)	39.07 ± 16.59 (11–76)**
Microcytosis	1.13 ± 0.99 (0–3)	1.07 ± 1.67 (0–5)	4.93 ± 5.99 (0–23)	9.13 ± 7.50 (1–26)**	22.53 ± 12.11 (2–46)**
Total cells analysed	15.154	15.120	15.092	15.123	15.307
Total cell changes (‰)	5.70	5.10	9.26*	12.36**	20.16**

Mean ± SD (minimum–maximum). Asterisks indicate significant differences at the 5% (*) and 1% (**) levels relative to control (Dunnett test).

**Figure 3:**
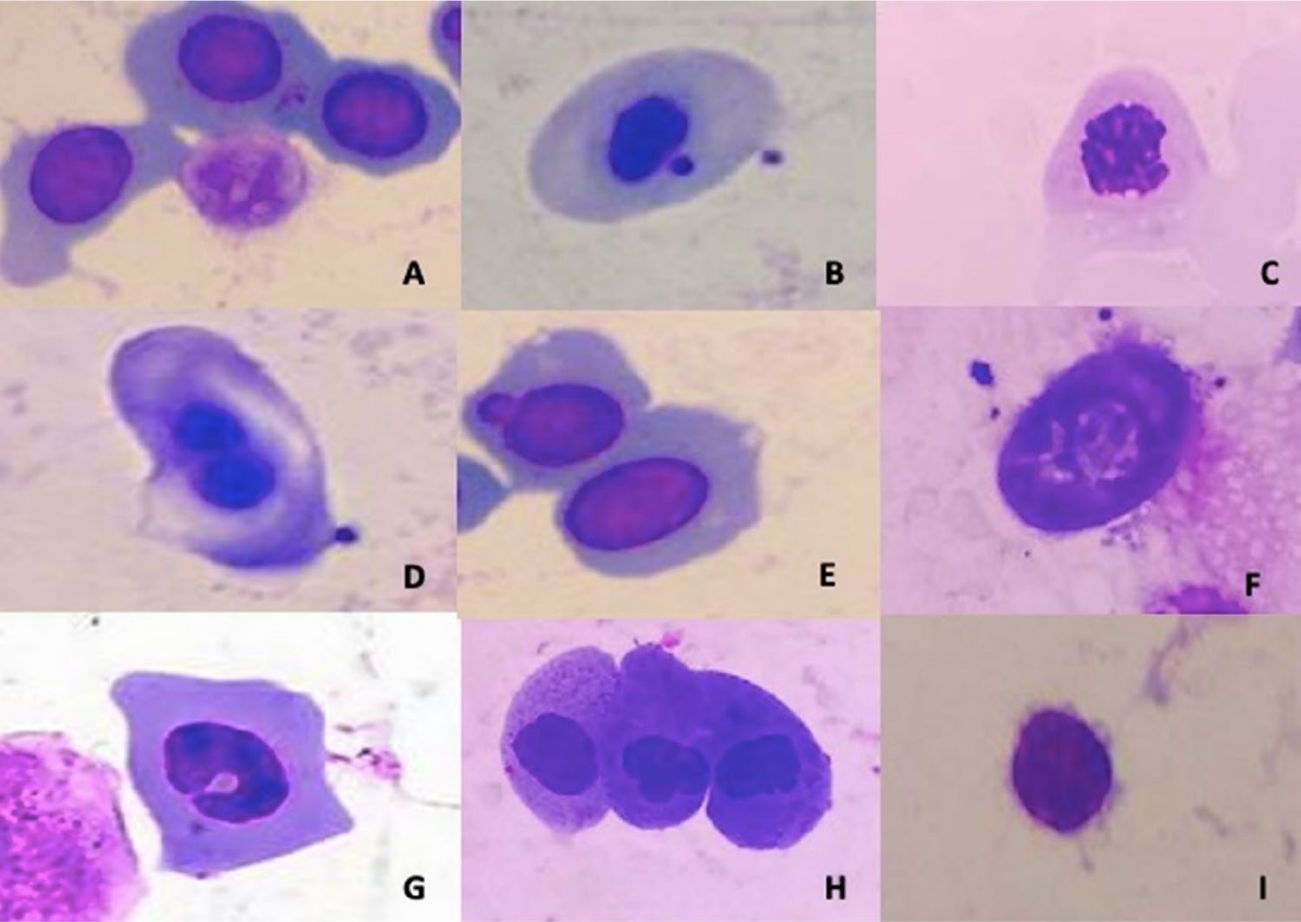
Micronucleus and erythrocyte nuclear abnormalities in *P. gracilis* tadpoles exposed to concentrations of the commercial formulation of cypermethrin. (**A**; control) cells with normal nuclei, (**B**) cell with micronucleus; (**C**) cells with apoptosis; (**D**) binucleated cell; (**E**) cell with bubble or bud; (**F**) cells with karyolysis; (**G**) notched cell; (**H**) lobed cells; (**I**) cells with microcytosis.

All tadpoles studies exhibited some nuclear abnormalities (Fig. 3), with 20.16 ‰ of abnormalities at the highest concentration (20 µg L^−1^) after 168 h of exposure. The most common ENAs at all concentrations was the notched nucleus (6.64‰ of the total cells analyzed), followed by a lobed nucleus (2.65‰) and a nucleus with bubble (1.17‰). Notched (F_4,70_ = 27.16; *p* < 0.05) and lobed cells (F_4,70_ = 13.94; *p* < 0.01) were significant at a dose of 3 µg L^−1^, while bubble cells (F_4,70_ = 38.04; *p* < 0.01) and microcytosis were significant from 6 µg L^−1^ (F_4,70_ = 24.47; *p* < 0.01) and apoptosis at the highest dose (20 µg L^−1^) (F_4,70_ = 11.82; *p* < 0.01) compared to control.

Total cell changes (‰) were significantly different from control treatment at concentrations of 3 µg L^−1^ (F_4;70_ = 53.4, *p* < 0.05), 6 µg L^−1^ (F_4;70_ = 53.4, *p* < 0.01), and 20 µg L^−1^ (F_4;70_ = 53.4, *p* < 0.01) (Table 1).

### Ecological risk analysis

Cypermethrin exhibited high acute (AHQ > 0.5) and chronic (CHQ > 1) ecological risk to *P. gracilis* for all variables evaluated (Table 2). The MATC value ranged from 1.73 to 10.95 μg L^−1^.

**Table 2:** Ecological risk assessment for tadpoles of *P. gracilis* exposed to the insecticide cypermethrin.

Test	LC_50_ (μg L^−1^)	NOEC (μg L^−1^)	LOEC (μg L^−1^)	MATC (μg L^−1^)	Endpoint	HQ
Acute	273.41	–	–	–	Mortality	0.71 _AHQ_
Chronic	–	3	6	4.24	Mortality	64.67_CHQ_
	–	1	3	1.73	MN + ENAs	97.00 _CHQ_
	–	3	6	4.24	Micronucleus	97.00 _CHQ_
	–	6	20	10.95	Apoptosis	14.92 _CHQ_
	–	3	6	4.24	Bubble	64.67 _CHQ_
	–	1	3	1.73	Notched	97.00 _CHQ_
	–	1	3	1.73	Lobed	97.00 _CHQ_
	–	3	6	4.24	Microcytosis	64.67 _CHQ_

Values for NOEC (no observed effect concentration), LOEC (lowest observed effect concentration), MATC (maximum acceptable toxicant concentration), and ecological risk (HQ) for micronucleus (MN) and other erythrocyte nuclear abnormalities (ENAs) observed in *P. gracilis*. *AHQ* acute hazard quotient, *CHQ*chronic hazard quotient.

## Discussion

Toxicity evaluation showed that the insecticide cypermethrin has a high acute toxicity to *P. gracilis*. The LC_50_ determined was 273.41 µg L^−1^, a value below 10,000 µg L^−1^, a reference value for the classification of the substance as very toxic according to GHS^34^. Similar results were also found for *P. cuvieri* (LC_50_ = 240 µg L^−1^)^28^ and lower for *P. biligonigerus* (LC_50_ = 129 µg L^−1^)^35^ and *Duttaphrynus. melanostictus* (LC_50_ = 3.34 µg L^−1^)^23^, indicating that cypermethrin can be lethal to several amphibian species. In addition, concentrations approaching those observed for several species have already been detected in natural waters (194 µg L^−1^)^21^, strongly suggesting that pesticides play an important role in the decline of populations of these aquatic species. Concentrations of cypermethrin considered sublethal or chronic also caused high mortality in *P. gracilis*. At a concentration of 20 µg L^−1^ cypermethrin, the mortality rate was more than 50% after a seven-day exposure to the insecticide. It is noteworthy that this result has characteristics of acute toxicity, since it caused the death of more than half of the cypermethrin-exposed individuals. Vanzetto et al.^7^ also confirmed the occurrence of changes in swimming activity and mouth morphology of *P. gracilis* tadpoles exposed to pyrethroids, affecting their ability to escape from predators and to feed, influencing the viability of anuran population^36,37^.

The observed high mortality was a consequence of genotoxic effects observed in amphibians exposed to different subconcentrations of cypermethrin (6 and 20 µg L^−1^), which was confirmed by the presence of micronuclei (MN) and erythrocyte nuclear abnormalities. The formation of MN indicates the occurrence of errors in mitotic division associated with weak binding of chromosomes to microtubules, a defect in the protein complex responsible for chromosome capture and transport, errors in chromosome segregation, and errors in DNA damage repair^38,39^ that may be associated with pesticide-induced oxidative stress^40,41^. Other abnormalities were also observed at all concentrations evaluated. Increasing cypermethrin concentrations resulted in a 5% and 20% increase in erythrocyte nuclear abnormalities at the lowest (1 µg L^−1^) and highest (20 µg L^−1^) doses assessed, respectively. Changes in species DNA, for example, can have significant effects on short- and long-term survival, declines in population size, changes in reproductive ability, inbreeding, loss of genetic diversity, and changes in migration rates. All of these factors affect species survival and conservation^42,43^. The formation of abnormalities in erythrocytes may indicate a blockade of cytokinesis leading to abnormal cell division (binucleated erythrocytes)^44,45^; lobed nuclei are protrusions of the nuclear membrane with multiple lobesarcau^46^, while other erythrocyte abnormalities may be related to DNA amplification, such as nuclear buds/bubbles^47^. The presence of anucleated erythrocytes may indicate effects on oxygen transport, especially in polluted waters^48,49^. While apoptosis is indicative of cell death^50^.

Other studies have also demonstrated the genotoxic effects of cypermethrin. Cabagna et al.^51^ demonstrated the presence of micronuclei and nuclear changes in cells such as binucleated cells and apoptotic cells in *Odontophrynus americanus* after exposure to high concentrations of cypermethrin (5000 and 10,000 µg L^−1^) for up to 96 h. Cypermethrin-induced apoptosis of cells was also detected in *P. biligonigerus*^52^ and *Rhinella arenarum*^53^. These results indicate that cypermethrin has a genotoxic effect on several species of aquatic organisms, and analysis of MN and ENAs may be an indicator of sublethal effects in amphibians and can be applied to native species and wild populations exposed to toxic substances^12^.

The commercial formulation of cypermethrin had a high ecological risk, both acute and chronic, with HQ above USEPA^54^ level of concern, and is likely to have adverse effects on species if present in the environment. In the chronic risk assessment, the NOEC for mortality was 3 µg L^−1^, confirming that concentrations already found in water may pose a risk to the species^55^. determined a NOEC of 500 µg L^−1^ for lethality of *R. arenarum* larvae after 168 h of exposure to a mixture of endosulfan and cypermethrin; this value was reduced to 0.0005 µg L^−1^ after 336 h. The authors show that the longer the exposure time, the lower the concentration that can have harmful effects on the species. It is also important to highlight that the NOEC value was higher than that observed for *P. gracilis* for the same exposure time, indicating species-specific responses to cypermethrin. Moreover, in terms of mortality, exposure of *P. gracilis* to cypermethrin caused a CHQ value of 64.67, a value higher than the reference value established by the USEPA^54^ and also reported for the larvae of *R. arenarum* (CHQ > 388.00 in 336 h), indicating that the insecticide studied poses a high risk to several amphibian species. Considering that *P. gracilis* completes metamorphosis in approximately 30 days^56^, it can be inferred that the cypermethrin concentrations tested can potentially promote population decline by preventing infected individuals from reaching the adult or reproductive stage at an early stage.

In the calculated risk assessment for micronuclei and other erythrocyte nuclear abnormalities, CHQ values ​​ranged from 14.92 to 97.00, suggesting that cypermethrin poses a potential genotoxic risk to *P. gracilis*, even in its natural habitat. The maximum acceptable concentration of the xenobiotic for *P. gracilis*, taking into account mortality, is 4.24 µg L^−1^. However, concentrations as low as 1 µg L^−1^ have shown genotoxic effects. This fact may lead to an increase in abnormal individuals^57^ and affect the development and reproduction of the species in its habitat, contributing to the decline of the amphibian population.

The commercial formulation of the insecticide cypermethrin showed high acute and chronic toxicity to *P. gracilis*. A high mortality rate was observed, possibly due to the toxic effects evidenced by the presence of micronuclei and erythrocyte nuclear abnormalities, especially notched nuclei, lobed nuclei, and nuclei with bubble. In addition, high ecological risk, both acute and chronic, was demonstrated for the species studied. These data combined with previous studies by our research group showing that even various commercial formulations of cypermethrin still cause a reduction in acetylcholinesterase (AChE) and butyrylcholinesterase (BchE) activities and oxidative stress^58^ and cause changes in swimming activity and mouth malformations^59^ in *P. gracilis* indicate the high lethal and sublethal toxicity of the commercial formulation of cypermethrin for this species. Hartmann et al.^60^ considered that commercial formulations of cypermethrin were most toxic to *P. gracilis* and another species of the same genus (*P. cuvieri*), compared to nine other pesticides. This suggests that environmentally relevant legally approved concentrations of cypermethrin may cause high mortality rates and contribute to long-term population declines.

Further studies are needed to evaluate the toxicity of the insecticide to amphibians, as the concentrations found in the environment caused high mortality rates and pose a potential risk to *P. gracilis*. Research with amphibian species should be encouraged because there are few data on these organisms, especially Brazilian species.

## Materials and methods

### Chemicals

Toxicity tests were conducted with the commercial formulation of the insecticide cypermethrin: Cyptrin 250 EC (Nufarm Indústria Química e Farmacêutica S.A., Maracanaú/CE, Brazil) consisting of 250 gL^−1^ of the active ingredient (a.i.) cypermethrin and 723 g L^−1^ of inert ingredients. The insecticide has a density of 1.12 g mL^−1^ at 22 °C, a melting point of 60–80 °C (pure isomers), a boiling point of 170–195 °C, and a vapor pressure of 4 × 10–8 mm Hg at 70 °C^61^. Under normal temperature and pH conditions, the product is stable to hydrolysis with a half-life greater than 50 days and stable to photolysis with a half-life greater than 100 days^62,63^.

The commercial formulation was used to prepare the stock solution (500 mg a.i.a. L^−1^), which was diluted in distilled water to obtain the concentrations to be used in the acute toxicity study: 100 µg L^−1^, 200 µg L^−1^^21^, 350 µg L^−1^, 400 µg L^−1^, 600 µg L^−1^, 700 µg L^−1^, and 800 µg a.i. L^−1^; and chronic toxicity: 1 µg L^−1^, 3 µg L^−1^^64^, 6 µg L^−1^^65^, and 20 µg a.i. L^−1^^66^.

### Test organism

Spawns of *Physalaemus gracilis* (Boulenger, 1833) were collected in natural environment in lakes in the city of Erechim, RS, Brazil (latitude: 27° 43′ 46.11″ South; longitude: 52° 16′ 54.40″ West), with egg laying lasting less than 24 h.

Immediately after collection, spawnings were placed in aquaria with a capacity of 15 L containing water with drinking water standards: temperature 22 °C ± 2 °C, pH 7.0 ± 0.5, dissolved oxygen 4.0 ± 1.0 mg L^−1^, turbidity < 5, conductivity 160 ± 10 µS cm^−1^, alkalinity (CaCO_3_) 9.74 mg L^−1^, Ca 6.76 mg L^−1^, Na 44.1 mg L^−1^, Mg 1.35 mg L^−1^, Fe 0.08 mg L^−1^, Ni < 0.001 mg L^−1^; under constant aeration and controlled laboratory conditions with temperature of 24 ± 2 °C, relative humidity between 60–80% and 12/12 h light–dark. Tadpoles were reared under these conditions until they reached developmental stage 25^67^. The tadpoles used in the tests had an average weight of 0.037 g ± 0.012 and an average length of 15.82 mm ± 1.59.

### Ethical approval and informed consent

The present study was approved by the Ethics Committee for the Animal Use (CEUA) of the Federal University of Fronteira Sul, Erechim, RS, Brazil, under protocol number 23205.003634/2017-70.

## Experimental design

### Toxicity tests

The acute toxicity test was performed on subjects exposed to six concentrations of the commercial formulation of cypermethrin: 100, 200, 350, 400, 600, 700, and 800 µg a.i. L^−1^ for 96 h. Water temperature (22 ± 2 °C) and dissolved oxygen (4 ± 2 mg L^−1^) were kept constant throughout the test period, and tadpole mortality was assessed every 24 h. Acute toxicity was classified according to the Globally Harmonized System of Classification and Labeling of Chemicals^34^ as: high toxicity (LC_50_ < 1 mg L^−1^), moderate toxicity (LC_50_ between 1 and 10 mg L^−1^), or low toxicity (LC_50_ > 10 mg L^−1^).

The chronic toxicity test lasted 168 h (7 days) under static conditions and using the sublethal concentrations: 1, 3, 6, and 20 µg a.i. L^−1^. In both tests, 10 tadpoles were evaluated per treatment with six replicates, resulting in a total of 60 individuals for each concentration. In parallel, a treatment was performed as a negative control using only water. Each experimental unit consisted of a sterile glass with a capacity of 500 mL and a density of 1 tadpole per 50 mL of solution. The flasks were covered with plastic film to prevent evaporation and were constantly aerated.

The present study was approved by the Ethics Committee for the Animal Use (CEUA) of the Federal University of Fronteira Sul, Erechim, RS, Brazil, under protocol number 23205.003634/2017-70. All methods were carried out in accordance with relevant guidelines and regulations. The study is reported in accordance with ARRIVE guidelines.

The water was chemically analyzed to determine pesticide concentrations at 0, 96, and 168 h. Analysis was performed at the Laboratory for Analysis of Pesticides (LARP), Federal University of Santa Maria, using gas chromatography coupled with triple quadrupole mass spectrometry (Varian model 1200, Palo Alto, CA, USA), according to Sabin et al.^68^ and Martins et al.^69^. The pesticide quantification in the water was shown as Supplementary material (Table SM1).

### Micronucleus and erythrocytes nuclear abnormalities

For micronucleus assay (MN) and erythrocyte nuclear abnormalities (ENAs), 15 tadpoles from each treatment were analyzed. Tadpoles were anesthetized with 5% lidocaine (50 mg g^−1^^70^, and blood samples were collected by cardiac puncture with disposable heparinized syringes. Blood smears were prepared on sterilized slides for microscopy, air dried, fixed with 100% methanol (4 °C) for 2 min, and then stained with 10% Giemsa solution in the dark for 15 min. At the end of the process, the slides were washed with distilled water to remove excess dye and dried at room temperature.

The presence of MN and ENAs was determined by analyzing at least 1000 erythrocytes from each tadpole using a 100 × microscope objective^71^. A total of 75,796 tadpole erythrocytes were evaluated considering cypermethrin concentrations and control. Genotoxicity was analyzed according to Carrasco et al. and Fenech et al.^38,72^ by determining the frequency of the following nuclear lesions: (1) anucleated cells: cells without nuclei; (2) apoptosis cells: fragmented nuclei, with cells programmed to die; (3) binucleated cells: cells with two nuclei; (4) nuclear bud or bubble cells: nucleus with a small protrusion of the nuclear membrane, bubble size similar to the size of micronuclei; (5) karyolysis cells: nucleus that has only the outline, without internal material; (6) notched cells: nuclei that have a well-defined slit or cut in their shape, also called kidney-shaped nuclei; (7) lobed cells: nuclei with protrusions larger than the bubbles described above; (8) cells with microcytosis: condensed nucleus and reduced cytoplasm; and. The changes were compared with the negative control results.

### Ecological risk analysis

Ecological risk was determined using the hazard quotient (HQ) according to the United States Environmental Protection Agency^54^. The HQ is calculated by the ratio between the estimated environmental concentration (EEC) and LC_50_ for acute risk (AHQ = EEC/LC_50_), and between ECC and no observed effect concentration (NOEC) for chronic risk (CHQ = EEC/NOEC). When it was not possible to statistically calculate NOEC, lowest observed effect concentration (LOEC) values were used to calculate HQ^73^. The HQ was compared to the level of concern (LOC—level of concern)^54^, where AHQ > 0.5 and CHQ > 1 indicate the existence of an ecological risk. The EEC is an estimated (or maximum) environmental contaminant concentration next to the geographic range of the species, using the maximum level of cypermethrin reported in Argentina (0.194 mg L^−1^)^25^. The maximum acceptable toxicant concentration (MATC) was calculated from the geometric mean of the LOEC and NOEC values.

### Statistical analysis

Acute toxicity test results (LC_50_) were analyzed by the TSK—Trimmed Spearman-Karber method^74^ using GBasic software. Chronic test data were previously tested for normality of errors (Shapiro Wilks) and homogeneity of variances (Bartlett). Results were analyzed with a one-way analysis of variance (ANOVA). Tukey’s test was used to compare data with each other, and Dunnett’s test was used to compare treatment data with the negative control.

LOEC and NOEC data were analyzed with the Dunnett test. Statistical tests were performed using Statistica 8.0 software (StatSoft) with a significance level of 95% (*p* < 0.05).

### Supplementary Information

Supplementary Table S1.

## Data Availability

Data are available in Supplementary Information and on request from the corresponding author.
