# Supplementary material for: Mortality and toxicity of a commercial formulation of cypermethrin in Physalaemus gracilis tadpoles
Source: Sci Rep. 2023 Oct 19;13:17826. doi: 10.1038/s41598-023-45090-7 (PMC10587170; doi:10.1038/s41598-023-45090-7)
Supplement: Supplementary file 1 — Supplementary Table S1. [file 41598_2023_45090_MOESM1_ESM.docx]

**MORTALITY AND TOXICITY OF A COMMERCIAL FORMULATION OF CYPERMETHRIN IN *PHYSALAEMUS GRACILIS* TADPOLES**

Natani Macagnan¹, Camila Fatima Rutkoski¹, Alexandre Folador^1^, Vrandrieli Jucieli Skovronski^1^, Caroline Müller¹, Paulo Afonso Hartmann¹, Marilia Hartmann¹*

**Table SM1.** Chemically analyzed to determine cypermethrin (CYP) concentrations at 0, 96, and 168 hours.**

| **Nominal Concentrations**  **(µg L^-1^)** | |  | | **CYP (µg L^-1^)** | |  |
| --- | --- | --- | --- | --- | --- | --- |
|  |  | **0h** | | **96h** | | **168h** |
| **1** | | 1.05 | | 0.95 | | 0.85 |
| **3** | | 3.03 | | 2.85 | | 2.55 |
| **6** | | 5.85 | | 5.38 | | 5.07 |
| **20** | | 19.85 | | 18.55 | | 16.7 |
|  |  | |  | |  |  |

**Limit of detection (LOD): 0.05 µg L^-1^; limit of quantitation: 0.21 µg L^-1^ (CYP)
